# Maternal Childhood Abuse Versus Neglect Associated with Differential Patterns of Infant Brain Development

**DOI:** 10.1007/s10802-023-01041-4

**Published:** 2023-05-09

**Authors:** Karlen Lyons-Ruth, Frances Haofei Li, Jennifer E. Khoury, Banu Ahtam, Michaela Sisitsky, Yangming Ou, Michelle Bosquet Enlow, Ellen Grant

**Affiliations:** 1 Department of Psychiatry, Harvard Medical School, Cambridge Hospital, 1493 Cambridge St., Cambridge, MA USA; 2 Department of Psychology, Mount Saint Vincent University, Halifax, NS Canada; 3 Department of Pediatrics, Harvard Medical School, Boston Children’s Hospital, Boston, MA USA; 4 Department of Psychiatry, Harvard Medical School, Boston Children’s Hospital, Boston, MA USA

**Keywords:** Maternal childhood maltreatment, Abuse, Neglect, Infant grey matter, Infant amygdala

## Abstract

Severity of maternal childhood maltreatment has been associated with lower infant grey matter volume and amygdala volume during the first two years of life. A developing literature argues that effects of threat (abuse) and of deprivation (neglect) should be assessed separately because these distinct aspects of adversity may have different impacts on developmental outcomes. However, distinct effects of threat versus deprivation have not been assessed in relation to intergenerational effects of child maltreatment. The objective of this study was to separately assess the links of maternal childhood abuse and neglect with infant grey matter volume (GMV), white matter volume (WMV), amygdala and hippocampal volume. Participants included 57 mother-infant dyads. Mothers were assessed for childhood abuse and neglect using the Adverse Childhood Experiences (ACE) questionnaire in a sample enriched for childhood maltreatment. Between 4 and 24 months (*M* age = 12.28 months, *SD* = 5.99), under natural sleep, infants completed an MRI using a 3.0 T Siemens scanner. GMV, WMV, amygdala and hippocampal volumes were extracted via automated segmentation. Maternal history of neglect, but not abuse, was associated with lower infant GMV. Maternal history of abuse, but not neglect, interacted with age such that abuse was associated with smaller infant amygdala volume at older ages. Results are consistent with a threat versus deprivation framework, in which threat impacts limbic regions central to the stress response, whereas deprivation impacts areas more central to cognitive function. Further studies are needed to identify mechanisms contributing to these differential intergenerational associations of threat versus deprivation.

The infant brain develops rapidly over the first two years of life, with grey-matter volume (GMV) reaching 80% of adult volume in that time (Gilmore et al., 2012; Groeschel et al., 2010). White-matter axonal connections also develop rapidly prenatally, with pruning and myelination largely beginning after birth (Dubois et al., 2014; Knickmeyer et al., 2008) and continuing to be refined into adulthood (Groeschel et al., 2010). Such periods of rapid growth are hypothesized to signal experience-expectant sensitivity to environmental inputs, which fosters early adaptation to the affordances in one’s environment (Turecki & Meaney, 2016).

Accumulating evidence suggests that effects of maltreatment in the mother’s childhood may be transmitted to the child, with intergenerational effects evident in the child’s social adaptation and mental health (e.g., Buss et al., 2017; Plant et al., 2018). There are empirical and theoretical reasons to hypothesize that maternal childhood maltreatment may influence offspring mental health risk in part through its effects on infant brain development (Buss et al., 2017; Khoury et al., 2021a; Moog et al., 2018). Therefore, a first step in addressing potential pathways of intergenerational transmission is to increase our understanding of the links between the mother’s childhood maltreatment and her infant’s brain development. Establishing such an association would provide empirical support for further examining potential mechanisms that may link maternal childhood maltreatment to infant brain development, such as gestational effects of maternal stress hormones (Buss et al., 2017; Moog et al., 2016) or postnatal disrupted caregiving behavior (Guyon-Harris et al., 2020; Khoury et al., 2021b). Mapping how infant brain development may be affected by maternal childhood maltreatment also facilitates the identification of child brain regions that may be involved in the further links to later social adaptation and mental health. Therefore, examining links between the mother’s own history of maltreatment and alterations in her infant’s brain development is one critical step toward identifying the multiple mechanisms likely to mediate intergenerational transmission of adversity.

Two previous studies have examined the severity of maternal childhood maltreatment in relation to infant brain volumes during the first two years of life. Moog et al. (2018) found that maternal childhood maltreatment was associated with reduced newborn GMV but not with newborn amygdala or hippocampal volume. Khoury et al. (2021a) found that severity of maternal childhood maltreatment was associated with reduced infant GMV from ages four to 24 months. In addition, right hemisphere amygdala volume was reduced in relation to severity of the mother’s childhood maltreatment, but only after 18 months of age (Khoury et al., 2021a).

The later onset of amygdala effects may represent evidence of an early hyporesponsive period to mother-associated threat, consistent with randomized rodent studies (Opendak & Sullivan, 2016). This early hyporesponsive period may serve to protect the infant’s bonding to the mother as an important survival mechanism, even in the face of maternal aversive behavior (Hostinar et al., 2014).

However, an increasingly prominent theoretical framework is positing that *direct experiences* of threat and of deprivation may have different influences on the developing brain (McLaughlin et al., 2014, 2019), raising the possibility that threat and deprivation also need to be separated in studies of intergenerational transmission. In this framework, threat involves harm or the potential for harm to the child, and includes experiences such as sexual abuse, physical or psychological abuse, witnessing domestic violence, and exposure to community violence. Such threat experiences are thought to have similar influences on emotional and neural development that will vary in relation to the severity of the threat experienced. In contrast, deprivation involves an absence of expected inputs from the environment, including nurturance and stimulation (Mclaughlin et al., 2019). Thus, threat and deprivation experiences are frequently operationalized as experiences of abuse and neglect, respectively. In a review of studies of children exposed to early adversity but assessed for brain volumes later in childhood, McLaughlin et al. (2019) found that direct experiences of threat but not deprivation were consistently linked to reduced amygdala volume, and less consistently to reduced hippocampal volume, as well as to heightened amygdala reactivity to threat (Cuartas et al., 2021; Shafer et al., 2022). In contrast, deprivation, but not threat, was more consistently associated with reductions in cortical grey matter thickness and alterations in frontoparietal circuits, which in turn are linked to differences in cognitive ability, associative and implicit learning, language skills, and executive functions (McLaughlin et al., 2017, 2019; Shafer et al., 2022).

These findings also have implications for differential transmission of threat and deprivation across generations. If the mother’s own childhood history of threat or deprivation differentially affects her own neurobiology, by either heightening her amygdala reactivity to threat or affecting circuits critical to executive function, these differing maternal outcomes may not result in a uniform intergenerational effect of adversity, but instead produce different effects on the infant’s developing brain. Transmission of these differential effects of threat versus deprivation in the mother’s childhood experience could occur through associated differences in both gestational maternal hormones (Buss et al., 2017) and postnatal maternal behavior (Guyon-Harris et al., 2020; Khoury et al., 2021b).

In addition, if maternal childhood history of threat and deprivation affect infant neurobiological development differently, then combining different forms of maternal adversity into a single index potentially risks underestimating or even failing to detect effects that are, in fact, present. Underestimating or failing to detect effects of adversity can undermine efforts to allocate societal resources toward ameliorating the effects of adversity. In addition, if the intergenerational effects of threat and deprivation are to some extent distinct, using an overall adversity index may lead to developmental interventions that are overly global and not adequately targeted to the outcomes associated with a particular form of adversity, e.g. targeting harsh parenting (threat) when lack of parental stimulation (deprivation) is more prevalent in the risk group of interest.

Identifying the neurobiological effects associated with abuse and neglect is critical given that worldwide prevalence rates of child maltreatment range between 12 and 36%, with neglect being more frequent than abuse (Stoltenborgh et al., 2015). In the U.S., for example, child neglect constituted 75% of child protective service reports (U.S. Department of Health & Human Services, Administration for Children and Families, Children’s Bureau, 2021). However, neglect is typically understudied compared to abuse. A meta-analysis identified 31 studies of neglect compared to more than 200 studies of sexual abuse (Stoltenborgh et al., 2015). Similarly, a meta-analysis of the parenting behaviors of maltreating parents found 18 studies of abuse but only eight studies of neglect (Wilson et al., 2008), with neglect associated with low parental involvement and abuse with harsh parenting. In both reviews, researchers called for more work to delineate neglect-related outcomes in children (Stoltenborgh et al., 2015; Wilson et al., 2008).

One difficulty in examining the potentially distinct effects of abuse and neglect is that abuse and neglect experiences overlap in a subset of children (Armour et al., 2014; Guyon-Harris et al., 2020; Khoury et al., 2021b; Pears et al., 2008). Thus, this overlap needs to be taken into account in research distinguishing threat and deprivation. One approach to differentiating effects associated with the two types of adversity is by assessing both types of adversity and controlling for effects of one type when assessing for effects of the other.

Direct effects of infancy risk factors not clearly fitting to a threat versus deprivation framework have also been studied as predictors of differences in infant or child brain volumes. Family risk factors associated with reduced GMV include lower socioeconomic status (Betancourt et al., 2016; Hanson et al., 2013) and lower parental sensitivity (Kok et al., 2015; Sethna et al., 2017), with neither factor associated with WMV (Betancourt et al., 2016; Hanson et al., 2013; Sethna et al., 2017). Other studies have examined associations between amygdala volumes later in childhood and maternal depression in infancy. Two studies found risk-related increases in amygdala volume (Lupien et al., 2011; Wen et al., 2017) and one found increase in overall subcortical grey matter (Sethna et al., 2021). No studies of maternal depression have reported hippocampal effects. Based on these findings, both SES and maternal depression were assessed as potential covariates in the current study.

As noted above, studies differentiating effects of threat and deprivation on the childhood brain have largely assessed brain development later in childhood rather than in infancy and have examined associations with direct experiences of threat and deprivation (Cuartas et al., 2021; Mclaughlin et al., 2017, 2019). Studies assessing differential effects of threat and deprivation have not yet evaluated whether this framework might also apply to the intergenerational transmission of the effects of *the mother’s* childhood maltreatment to her infant. The current study examines whether the mother’s childhood experiences of threat versus deprivation in her own childhood have differential effects on her infant’s neurobiological development during the first two years of life.

## Aims and Hypotheses

The present report is based on the infant sample reported on by Khoury et al. (2021a). The current study builds on this prior work by focusing on the potentially distinct effects of maternal history of threat (abuse) versus deprivation (neglect) in relation to infant brain volumes, including GMV, amygdala volume, hippocampal volume, and WMV. GMV and amygdala volume are of particular interest because cortical grey matter and amygdala volumes have been shown to be differentially sensitive to deprivation versus threat, respectively, when directly experienced by the individual (Mclaughlin et al., 2019).

In the current study, we operationalized threat as experiences of sexual, physical, or emotional abuse and deprivation as experiences of childhood physical or emotional neglect in the mother’s childhood. We advanced two hypotheses: 1) mothers’ experience of deprivation would be differentially associated with reductions in infant GMV, and 2) mothers’ experience of threat would be differentially associated with reductions in infant amygdala, as a central structure in the threat detection network. Based on less consistent evidence, hippocampal volume was also tentatively expected to be reduced in relation to threat. Overall WMV was also assessed on an exploratory basis to evaluate the specificity of threat/deprivation effects. Given the lack of evidence linking WMV to either threat or deprivation, no effects were hypothesized. Based on rapid brain growth in infancy and on evidence of a potential hyporesponsive period for limbic responding in early infancy (Opendak & Sullivan, 2016), all models assessed whether infant age moderated relations between maternal threat/deprivation and infant brain volumes.

## Methods and Materials

### Participants and Procedures

Fifty-seven mother-infant dyads were included in the current analyses. Dyads were drawn from a cohort of 181 families enrolled in the Mother-Infant Neurobiological Development (MIND) study, a longitudinal study designed to assess the effects of maternal childhood maltreatment on maternal and infant behaviors and neurobiological markers over the first two years of life. Mothers were recruited through prenatal classes, community flyers, and local birth records and screened for study eligibility via the Adverse Childhood Experiences (ACE) questionnaire (Felitti et al., 1998). The sample was stratified such that approximately half of the mothers had experienced maltreatment. Exclusion criteria were: 1) English not a primary language spoken at home, 2) maternal age ≥ 44 years at infant birth, 3) infant < 36 weeks gestation or < 2500 g at birth, and 4) infant congenital defect or disorder. Exclusion criteria 2–4 were included to reduce potential sources of risk for atypical infant development due to factors not of interest in the present study. This study was approved by the Institutional Review Board [Partners Healthcare IRB Protocol #: 2014P002522]. Mothers provided written informed consent before initiation of study activities.

Mother-infant dyads participated in behavioral assessments at 4 months and 15 months. Upon study entry, the first half of participating families were offered infant MRI scans after the 15-month assessment, and the second half of participants were offered scans after the 4-month assessment. Thirty-one infants (16.58%) declined to participate in the MRI assessment when offered, and thirty-one (16.58%) withdrew from the MRI assessment prior to their scheduled visit or were not reachable for scheduling. Of the 119 families who were scheduled for the MRI, 52.10% (n = 62) attempted to complete the MRI but were unsuccessful. Among those 62 infants, unsuccessful scans occurred because the infant a) did not fall asleep before the scan (n = 23), b) woke up during the transfer to the scanner or during the scan (n = 30), c) moved too much during the scan to produce usable data (n = 7), or d) could not tolerate the ear protection or head coil (n = 2). This resulted in 57 infants successfully completing the MRI (*M*age in days = 357.93, *SD* = 185.98, Range 122–750 days or 4–24 months). MRI participants did not differ from MRI non-participants on any key study variables (infant sex, race, ethnicity, annual household income, maternal education, depressive symptoms, or severity of maltreatment on the ACE, *p*_range_ = 0.45–0.77).

Age at scan was distributed along a continuum from age 4 months to 24 months, due to the often long lead times and frequent rescheduling needed to conduct scans at night during infant sleep. Variations in age at scan were not correlated with infant sex, minority status, family income, maternal education, or maternal depression scores, *r*’s = -0.07–0.13, all *p*’s ns. Age at scan was negatively associated with gestational age, r = -0.31, p = 0.05, but this relation to gestational age was hard to interpret because of the limited range of gestational age (36 weeks to 42 weeks) in this sample, due to exclusion criteria for gestation less than 36 weeks (see Table 1). There was also a moderate association between older ages at scan and lower ACE scores (*r* = -0.35, *p* < 0.01), reflecting the difficulty in obtaining successful scans from at-risk toddlers. To control for this association, age at scan was included as a main effect in all analyses and moderation by age was assessed in relation to all maltreatment effects on brain volumes.

**Table 1:** Sociodemographic characteristics and descriptive statistics

	**M (SD)/% (N)**	**Range**
Gestational age (weeks) ^a^	*M* = 39.48 (*SD* = 1.60)	36–42
Infant age (days) at MRI	*M* = 357.93 (*SD* = 185.98)	122–750
Infant race/ethnicity		
White/Non-Hispanic	57.9% (n = 33)	–
Black	8.8% (n = 5)	–
Asian	1.8% (n = 1)	
Hispanic	5.3% (n = 3)	
Multi-racial	26.3% (n = 15)	
Maternal education		
High school	14.0% (n = 8)	–
Associate degree	8.8% (n = 5)	–
Bachelor’s degree	24.6% (n = 14)	–
Master’s degree	35.1% (n = 20)	–
Doctoral degree	17.5%(n = 10)	–
Annual household income		
$0 to $15,000	7.0% (n = 4)	–
$16,000 to $25,000	3.5% (n = 2)	–
$26,000 to $50,000	10.5% (n = 6)	–
$51,000 to $75,000	26.3% (n = 15)	–
$76,000 to $100,000	17.5% (n = 10)	–
$101,000 to $150,000	17.5% (n = 10)	–
$151,000 +	17.5% (n = 10)	–
ACE severity of neglect	M = 0.39 (SD = 0.67)	0–2
ACE severity of abuse	M = 0.98 (SD = 1.08)	0–3
Grey matter volume	M = 541885.60 (SD = 117789.69)	294497–737526
White matter volume	M = 355680.70 (SD = 82719.53)	173861–521082
Amygdala		
Right amygdala	M = 1253.47 (SD = 436.85)	621–2754
Left amygdala	M = 1133.89 (SD = 329.91)	603–2041
Hippocampus		
Right hippocampus	M = 2953.68 (SD = 630.90)	1784–4642
Left hippocampus	M = 3145.86 (SD = 643.06)	1875–5438

Brain volume metric is mm^3^. N = 57, except for regions with outliers removed, resulting in right and left amygdala volume N = 56; right hippocampal volume N = 56; left hippocampal volume N = 55

*ACE* Adverse Childhood Experiences, *MRI* magnetic resonance imaging

^a^Infants below 36 weeks gestation at birth were excluded from study

### Measures

#### Maternal Experiences of Childhood Maltreatment

Mothers completed the 10-item ACE questionnaire (Felitti et al., 1998) during an initial screening call. The ACE is valid in relation to infant developmental outcomes (Racine et al., 2018), as well as to adult medical morbidity and mortality (Felitti et al., 1998). The ACE also correlates highly with more detailed assessments of childhood maltreatment (Teicher & Parigger, 2015). The 10-item ACE had good reliability (α = 0.79, N = 57) in the current sample. Most relevant to the current study, five items on the ACE ask about the presence of emotional, physical, and sexual abuse, and emotional and physical neglect. The three abuse items were summed to yield a score ranging from 0–3 (MCAbuse severity), and the two neglect items were summed to yield a score ranging from 0–2 (MCNeglect severity).

#### Sociodemographic Questionnaire

Sociodemographic characteristics were assessed by maternal interview, including infant sex, infant gestational age at birth (in weeks), family income, maternal education, and infant race/ethnicity. Given that most infants from racial or ethnic minority groups were multiracial/multiethnic (Table 1), a dichotomous variable denoting infant minority/non-minority racial/ethnic status was created and used in analyses.

#### Maternal Depressive Symptoms

Maternal depressive symptoms were assessed using the Edinburgh Postnatal Depression Scale (EPDS; Cox et al., 1987) at 4 months infant age. The EPDS consists of 10 items (total score range 0 to 30) and is widely used for screening for postpartum depression (Cox et al., 1987). The current sample had good reliability (α = 0.86).

### Imaging Data Acquisition and Processing

Infant MRIs were performed on a 3.0 T Siemens Skyra scanner, with a 64-channel head coil. Infants were scanned during natural sleep, with no sedation. The T_1_-weighted acquisition used an advanced version of the Magnetization Prepared Rapid Acquisition Gradient Echo (MPRAGE) sequence, where fast, low resolution volumetric navigators were played each repetition period and were used for prospective motion correction (Tisdall et al., 2012). The specific imaging parameters of the MPRAGE acquisition included: voxel size = 1 × 1 × 1 mm^3^, repetition time (TR) = 2500–2540 ms, echo time (TE) = 1.65–2.37 ms, inversion time (TI) = 1450–1470 ms, field of view (FOV) = 192 × 192 mm^2^ and between 144–173 slices, enough to cover the entire brain of the infant. After performing visual quality control using the Freeview software (surfer.nmr.mgh.harvard.edu), the T_1_-weighted volumes were manually aligned along the AC-PC plane, and underwent N4 bias correction (Tustison et al., 2010), field of view normalization (Ou et al., 2018) and multi-atlas skull stripping (Doshi et al., 2013). These steps were followed by automatic segmentation into cortical and cerebellar gray and white matter as well as subcortical gray matter regions, including amygdala and hippocampus, using a multi-atlas-to-subject registration and fusion (Doshi et al., 2016), that have been validated (Ou et al., 2011, 2014) and adapted to infant brain MRIs (Morton et al., 2020; Ou et al., 2017). Quality control of the segmentations was visually performed using the FslView software (https://fsl.fmrib.ox.ac.uk/). These segmentations enabled the extraction of GMV (total of cortical, cerebellar and deep gray matter) and WMV (total of cerebral and cerebellar white matter). In addition, the right and left hemisphere amygdala and hippocampal volumes were extracted from the deep gray volumes.

These steps were followed by automatic segmentation into cortical and subcortical regions as well as tissue types classification using a multi-atlas-to-subject registration and fusion (Doshi et al., 2016) that have been validated (Ou et al., 2011, 2014) and adapted to infant brain MRIs (Morton et al., 2020; Ou et al., 2017). Quality control of the segmentations was visually performed using the FslView software (https://fsl.fmrib.ox.ac.uk/). These segmentations enabled the extraction of GMV and WMV, as well as right and left hemisphere amygdala and hippocampal volumes.

### Statistical Analyses

Statistical analyses were conducted using IBM SPSS Statistics 26 and RStudio (Version 1.3.1056). Linear regressions were conducted using maximum likelihood estimation with robust standard errors (MLR) and full information maximum likelihood (FIML) to account for missing data via the SEM function in the Lavaan package (Rosseel, 2012). Outliers ≥ 3 SD from the mean of brain volumes were removed and missing data were estimated using FIML to retain participants. Two infants had outliers: one infant for left and right amygdala and left hippocampal volumes, and one infant for left and right hippocampus, which were set to missing for analyses. There were no missing data points on other variables in the models. Based on Little's MCAR test, both amygdala and hippocampal volumes were missing at random, so that data were appropriate for use of FIML [amygdala: *X*^2^ (9) = 10.29, *p* = 0.33; hippocampus: *X*^2^ (19) = 29.185, *p* = 0.06].^1^ First, sociodemographic variables and depressive symptoms were assessed for significant bivariate associations with brain regions, and any variables with significant correlations were included as covariates in the relevant models. Regression models were run with each brain region as the dependent variable (GMV, WMV, and left and right amygdala and hippocampal volumes), and with MCAbuse severity, MCNeglect severity, and infant age at assessment as independent variables. Both MCNeglect severity and MCAbuse severity were entered into the same models to account for expected overlap between mother’s experiences of childhood abuse and neglect. Two additional models for each region assessed potential age moderation and included the above independent variables as well as 1) MCAbuse severity by age or 2) MCNeglect severity by age.^2^ Power analyses were computed to assess the effect sizes that could be detected in our models with N = 57 at 0.80 power. Based on the effect size benchmarks where *f*^2^ ≥ 0.02, *f*^2^ ≥ 0.15, and *f*^2^ ≥ 0.35 represent small, medium, and large effect sizes, respectively (Cohen, 1988; Selya et al., 2012), regression models with 4 or 5 predictors were powered to detect the incremental effect of a single variable of interest, significant at *p* = 0.05 two-tailed, with effect sizes ranging from *f*^2^ = 0.1430 to 0.1431 (Faul et al., 2007). Thus, analyses were powered to detect medium effects sizes but were not well-powered to detect small effect sizes.

## Results

### Descriptives and Covariates

**Fig. 1:**
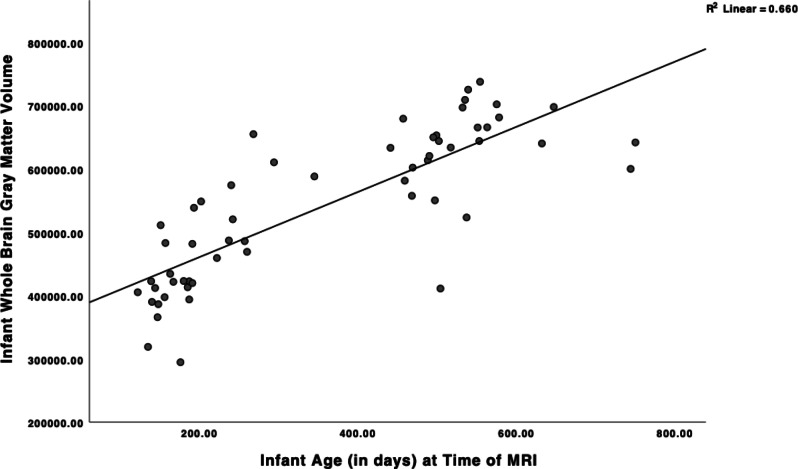
Scatterplot displaying the ages of infant MRI assessments and the association with infant grey matter volume. Note. Age in days: *M* = 357.93; *SD* = 185.98; 49% male. *N* = 57; Brain volume metric is mm^3^

Table 1 displays descriptive statistics for all study variables. Childhood maltreatment was endorsed by 58% of mothers, with 54.4% (n = 31) experiencing one or more forms of abuse, 28.1% (n = 16) experiencing one or more forms of neglect, and 28.1% (n = 16) experiencing both forms of maltreatment (categories not mutually exclusive). Infant age at MRI was strongly positively correlated with GMV (*r* = 0.81, *p* < 0.001; Fig. 1), but not other brain volumes (*r*_range_ = 0.13 to 0.20, all *p* n.s), consistent with the rapid growth in GMV over the first two years of life (Gilmore et al., 2012). Potential covariates, including annual household income, maternal education, maternal depressive symptoms, infant sex, infant minority status, and gestational age at birth were assessed in relation to infant brain volumes. Higher maternal depressive symptoms were associated with larger infant left hippocampal volume (*r* = 0.28, *p* < 0.05). No other potential covariates were associated with infant brain volumes (*r*_range_ = -0.04–0.25, all n.s.). Although sex was not significantly associated with brain volumes, given sex effects documented at older ages (Gilmore et al., 2018), sex was controlled in all analyses. Thus, all analyses included infant age at MRI and infant sex. In addition, depression was included as a covariate in left hippocampal analyses. Lastly, due to rapid brain growth over this age range, all models assessed whether the relation between type of maltreatment and brain volume was moderated by age.

### Maternal Childhood Neglect and Abuse in Relation to Infant Brain Volumes

#### Infant GMV

**Fig. 2:**
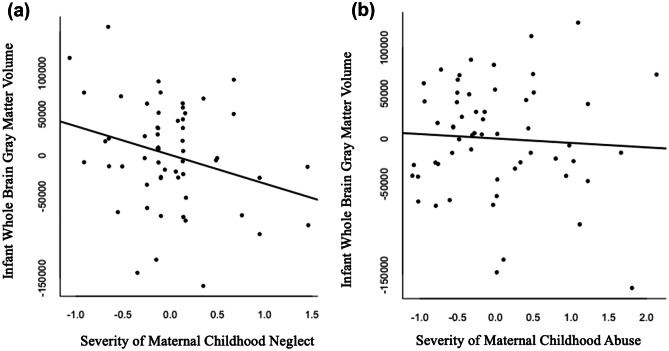
Infant grey matter volume in relation to severity of maternal childhood neglect (**a**) and abuse (**b**). Note. N = 57. Residual values for GMV after controlling for age, sex, and the other type of maltreatment (abuse or neglect). Y axis indicates deviation from GMV mean score; X axis indicates deviation from ACE mean score. Maternal childhood neglect *β* = -0.20, SE = 0.09, 95% CI [-0.38, -0.02]; maternal childhood abuse *β* = -0.05, SE = 0.10, 95% CI -0.23 – 0.14]

To evaluate the independent contributions of MCNeglect severity and MCAbuse severity to infant GMV, the regression model included both MCNeglect severity and MCAbuse severity, with infant age and sex controlled. Consistent with Hypothesis 1, when both types of maltreatment were included in the same model, only MCNeglect accounted for significant variance in infant GMV (see Table 2). These results indicate that severity of maternal childhood neglect made an independent contribution to reduced infant GMV, whereas maternal childhood abuse did not explain additional variability in infant GMV with neglect controlled. Neither the MCNeglect by age interaction nor the MCAbuse interaction terms were significant for GMV (Table 2). These findings suggest that the association of MCNeglect severity with lower GMV was similar across the first two years of life. The slopes of the effects of maternal childhood neglect and abuse on residualized infant GMV are shown in Fig. 2a, b.

**Table 2:** Infant brain volumes in relation to severity of maternal childhood neglect and severity of maternal childhood abuse

Dependent Variable	Effect	Unstd beta	*β*	*SE*	95% CI		*p*
					*LL*	*UL*	
**GMV**	**Main Effects Model:**						
	MCAbuse	-4.88	-0.05	0.10	-0.23	0.14	0.64
	MCNeglect	-35.03	-0.20	0.09	-0.38	-0.02	0.03
	Age	48.45	0.77	0.06	0.65	0.88	0.00
	Sex	29.07	0.12	0.07	-0.02	0.27	0.08
	**Interaction Term:**^**a**^						
	MCNeglect * age *β* = -0.04, SE = 0.14, CIs = -0.32, 0.24						
	**Interaction Term:**^**a**^						
	MCAbuse * age *β* = -0.23, SE = 0.15, CIs = -0.52, 0.06						
**Right amygdala**	**Main Effects Model:**						
	MCAbuse	0.00	0.01	0.18	-0.35	0.36	0.97
	MCNeglect	0.01	0.01	0.17	-0.33	0.35	0.95
	Age	0.04	0.15	0.14	-0.13	0.43	0.29
	Sex	0.12	0.14	0.14	-0.13	0.41	0.30
	**Interaction Term:**^**a**^						
	MCNeglect*age *β* = -0.36, SE = 0.26, CIs = -0.87, 0.16						
	**Interaction Term:**^**a**^						
	MCAbuse*age *β* = -0.61, SE = 0.27, CIs = -1.13, -0.09, p < 0.05						
**Left amygdala**	**Main Effects Model:**						
	MCAbuse	-0.03	-0.09	0.18	-0.43	0.26	0.63
	MCNeglect	0.03	0.07	0.17	-0.26	0.39	0.70
	Age	0.03	0.18	0.14	-0.09	0.45	0.19
	Sex	0.17	0.26	0.13	0.01	0.51	0.05
	**Interaction Term:**^**a**^						
	MCNeglect*age *β* = 0.01, SE = 0.26, CIs = -0.51, 0.53						
	**Interaction Term:**^**a**^						
	MCAbuse*age *β* = -0.37, SE = 0.27, CIs = -0.90, 0.16						
**Left Hippocamp**	**Main Effects Model:**						
	MCAbuse	0.11	0.19	0.17	-0.14	0.52	0.25
	MCNeglect	-0.20	-0.21	0.17	-0.53	0.12	0.21
	Age	0.08	0.24	0.13	-0.02	0.49	0.07
	Sex	0.26	0.20	0.13	-0.04	0.45	0.11
	Mat. dep	0.45	0.33	0.13	0.08	0.58	0.01
	**Interaction Term:**^**a**^						
	MCNeglect*age *β* = 0.01, SE = 0.25, CIs = -0.48, 0.50						
	**Interaction Term:**^**a**^						
	MCAbuse*age *β* = 0.06, SE = 0.27, CIs = -0.46, 0.59						
**Right Hippocamp**	**Main Effects Model:**						
	MCAbuse	-0.00	-0.00	0.18	-0.36	0.35	0.98
	MCNeglect	0.01	0.01	0.17	-0.33	0.34	0.97
	Age	0.07	0.18	0.14	-0.09	0.45	0.20
	Sex	0.29	0.21	0.13	-0.05	0.46	0.12
	**Interaction Term:**^**a**^						
	MCNeglect*age *β* = -0.29, SE = 0.26, CIs = -0.81, 0.22						
	**Interaction Term:**^**a**^						
	MCAbuse*age *β* = -0.24, SE = 0.28, CIs = -0.78, 0.31						
**WMV**	**Main Effects Model:**						
	MCAbuse	-20.79	-0.27	0.17	-0.61	0.07	0.12
	MCNeglect	19.24	0.16	0.17	-0.17	0.48	0.35
	Age	3.61	0.08	0.14	-0.19	0.35	0.56
	Sex	32.92	0.20	0.13	-0.05	0.46	0.12
	**Interaction Term:**^**a**^						
	MCNeglect*age *β* = -0.36, SE = 0.26, CIs = -0.87, 0.14						
	**Interaction Term:**^**a**^						
	MCAbuse*age *β* = -0.50, SE = 0.27, CIs = -1.02, 0.03						

N = 57

All main effects are shown as calculated before entry of interaction term for age by type of maltreatment

*Unstd beta* Unstandardized beta, *MCAbuse* Severity of maternal childhood abuse, *MCNeglect* Severity of maternal childhood neglect, *GMV* Grey matter volume, *WMV* White matter volume, *Hippocamp* hippocampus volume, *Mat. dep.* maternal depression

^a^Only one interaction term was entered per model, given limited sample size

#### Infant Amygdala Volume

**Fig. 3:**
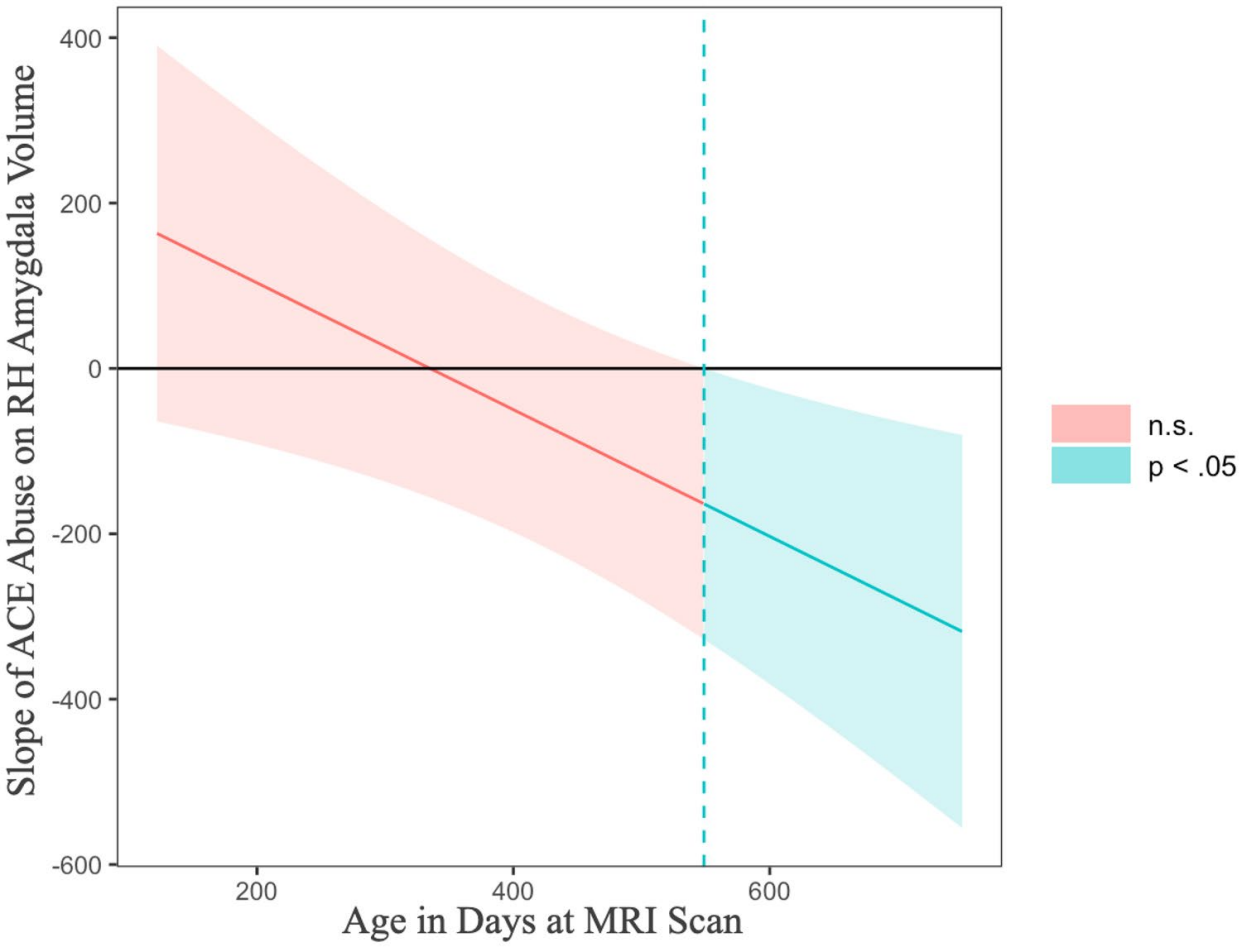
Region of significance of the interaction between severity of maternal childhood abuse and infant age at MRI on infant right hemisphere amygdala volume. Note. Region of significance plotted using the Johnson-Neyman method (Johnson & Neyman, 1936). Graph derived using linear regression with MLR estimation, without FIML. N = 56 (one outlier removed). When infant age is outside the interval [-102.35, 548.65 days], or 18 months, the slope is significant, p < 0.05

Using similar regression models for infant right amygdala volume, there was no main effect of MCNeglect severity nor interaction effect of MCNeglect severity by infant age (Table 2). However, consistent with hypotheses, there was a significant interaction between MCAbuse severity and infant age, such that MCAbuse severity was associated with reduced infant right amygdala volume at older ages (Table 2). Analysis of the region of significance for the age by MCAbuse interaction indicated that infant right amygdala volume was reduced in relation to MCAbuse severity after 18.0 months of age (older than 548.65 days; Johnson & Neyman, 1936; see Fig. 3).

For infant left amygdala volume, there was no main effect of either MCNeglect severity or MCAbuse severity and no moderation by age for either variable (Table 2).

#### Infant Hippocampal Volume

There were no significant effects of MCNeglect severity or MCAbuse severity on infant right or left hippocampal volumes, nor were there any significant interaction effects with age (Table 2). Higher maternal depressive symptoms remained associated with larger left hippocampal volumes with other variables controlled (Table 2).

#### Infant WMV

Consistent with prior literature, no effects of MCNeglect severity, MCAbuse severity, or interactions of these variables with infant age were found in relation to infant WMV (Table 2).

## Discussion

Consistent with a threat versus deprivation framework, the current study provides evidence for differential associations of maternal childhood abuse and neglect with different patterns of infant brain development during the first two years of life. First, maternal experience of neglect was uniquely associated with lower infant GMV, whereas maternal experience of abuse was not. Notably, these results are consistent with a threat versus deprivation framework, which hypothesizes that deficits in brain regions associated with cognitive and executive functioning will result from experiences of deprivation. The current findings are unique, however, in finding this effect at the level of intergenerational transmission, where the mother’s experiences of deprivation in childhood are associated with lower GMV in her infant. The threat versus deprivation framework also suggests that these neural deficits will be associated with later cognitive deficits in such areas as language and executive functioning (McLaughlin et al., 2019). Thus, it will be important in future work to assess these functions in relation to reduced GMV as children age.

Notably, the association between maternal childhood neglect and reduced infant GMV was not moderated by age at MRI, suggesting that the effect was similar over the first two years of life. This finding of an early effect of mother’s childhood maltreatment on infant brain development is consistent with work by Moog et al. (2018) who found that maternal childhood maltreatment was associated with lower GMV among newborns. Differential effects of maternal childhood abuse and neglect were not evaluated in that study, but the current results suggest a greater effect of maternal childhood neglect on GMV. Notably, GMV increases rapidly during the first two years of life (Knickmeyer et al., 2008), with relatively small increases thereafter. Thus, the reductions seen here may not be remediated in subsequent development, a possibility that needs exploration in future work.

In contrast, maternal childhood abuse was uniquely associated with smaller volume of right amygdala, with both childhood abuse and neglect in the model. This association of maternal childhood abuse with reduction in infant right amygdala volume is also consistent with a threat versus deprivation framework, which predicts that threat of attack or injury, as in experiences of abuse, will have particular impact on the threat response network, in which the amygdala plays a central role. In the current data, age moderated the effect of MCAbuse severity on right amygdala volume, such that there was an increasing negative association between severity of mother’s childhood abuse and amygdala volume with age, an association that became significant at approximately 18 months (Fig. 3). Consistent with the later onset of amygdala effects found here, Moog et al. (2018) did not find a significant association between overall severity of maternal childhood maltreatment and *newborn* amygdala volume. Thus, associations between mother’s childhood abuse and amygdala volume may not be evident in early infancy but may become detectable during the period of rapid amygdala growth that occurs over the early years of life. Consistent with this finding, Opendak and Sullivan (2016) found that rodent pups have a hyporesponsive period in early development to stressors associated with the mother. They speculate that this hyporesponsive period ensures bonding to the mother during the critical period for attachment formation. Further work is needed to assess whether there may be a similar hyporesponsive period during the first years of life among human infants that protects the developing mother-infant attachment bond.

In contrast, no effects of maternal childhood abuse or neglect were observed in relation to infant hippocampal volumes. However, there was an association between higher maternal depressive symptoms and reduced left hippocampal volume. Because previous work has not found links between child hippocampal volume and maternal depression (Lupien et al., 2011; Sethna et al., 2021; Wen et al., 2017), this finding needs replication in future studies.

There were no observed effects of either form of maternal childhood maltreatment on WMV, consistent with prior research that has failed to find links between mother’s childhood maltreatment (Moog et al., 2018) or other psychosocial risk factors (Hanson et al., 2013) and infant WMV. This may be due to the faster rate of growth in GMV in the first two years of life compared to WMV (Gilmore et al., 2018), as well as the long developmental course of white matter organization and myelination. However, studies that assess other aspects of white matter maturation, including resting state functional connectivity and integrity of white matter tracts, will be needed for more definitive assessment of differential effects of mother’s maltreatment history on early white matter development.

Results of the study add important specificity to previous work linking *overall* maternal childhood maltreatment to reductions in infant GMV and amygdala volumes (Khoury et al., 2021a; Moog et al., 2018). Current results also extend the growing literature on distinct effects of threat versus deprivation on the developing child (McLaughlin et al., 2019). To our knowledge, this is the first report to find differential associations of maternal childhood neglect and abuse with theoretically predicted infant brain volumes, suggesting differential intergenerational effects of maternal experiences of threat and deprivation. Second, most studies of threat versus deprivation have involved direct effects of threat or deprivation on brain development among older children (e.g. Cuartas et al., 2021; McLaughlin et al., 2019). In the current study, differential effects of maternal threat and deprivation on infant brain development were evident within the first two years of life. Finally, deprivation in most previous work has focused on extreme deprivation associated with institutional rearing. The current findings extend the literature by showing reduced GMV among infants reared at home by mothers with a history of childhood neglect.

Studies of the intergenerational transmission of overall adversity and studies of distinct neurobiological effects associated with threat versus deprivation have developed as separate literatures to date. We are aware of no study assessing differential links between maternal experiences of threat versus deprivation and development of specific brain regions in her infant. Thus, potential mechanisms that might contribute to such differential transmission must remain speculative. However, growing evidence links overall maternal childhood maltreatment with alterations in gene expression, maternal and infant gestational placental-fetal biology, and the child’s postnatal environment, all of which are likely to play a role in intergenerational transmission of adversity (Buss et al., 2017; Scorza et al., 2019). Lower infant grey matter volume has been shown to be present at birth in relation to overall maltreatment (Moog et al., 2018) and, in the current study, lower infant grey matter volume by four months of age was associated specifically with maternal experiences of childhood neglect. Thus, gestational mechanisms are strongly suggested as potential contributors to the differential effect of maternal experiences of neglect on lower infant grey matter volume. Moog et al. (2016) have also shown that maternal childhood maltreatment is related to differences in maternal-placental-fetal biology, specifically to production of placental corticotrophin-releasing hormone, which would be expected to affect fetal neurodevelopment. How such differences in the intrauterine environment might differ in relation to mother’s exposure to abuse versus neglect is not known. In addition, potential indicators of early postnatal neglect, including low SES and institutional rearing (Hanson et al., 2013; Mclaughlin et al., 2017) have been associated with lower overall grey matter volume or decreased cortical grey matter thickness. Thus, processes at the level of preconceptional epigenetic markers, gestational environment, and postnatal care are all likely to play a role in differential intergenerational effects of maternal childhood abuse and neglect on infant brain development.

Alterations in the mother’s neuroendocrine functioning may also affect infant brain development postnatally. Both blunted (downregulated) and elevated cortisol levels have been associated with childhood maltreatment (e.g., Bernard et al., 2017; Khoury et al., 2019). Thus, both down-regulation and up-regulation of the mother’s stress response system should be examined as possible postnatal influences on maternal regulation of the developing infant stress response system (Hostinar et al., 2014), with further potential effects on the development of stress-sensitive infant brain regions such as the amygdala.

### Limitations and Future Directions

Study limitations are important to note. First, the sample size is relatively small, so that findings must be considered preliminary and need to be replicated in larger samples. Second, the co-occurrence of experiences of abuse and neglect, here as in other studies of maternal history of maltreatment (e.g., Guyon-Harris et al., 2020; Khoury et al., 2021b), makes it difficult to evaluate the relative contributions of each form of maltreatment. While the statistical analyses controlled for each type of maltreatment when assessing the other type, additional work is needed to replicate these findings. Third, infant brain volumes were assessed cross-sectionally over a wide age range. Future work should examine longitudinal trajectories of infant brain growth related to maternal childhood abuse and neglect histories, in order to assess the replicability of these effects and to better understand potential change over the first two years in how maternal childhood maltreatment is linked to intergenerational effects on specific infant brain regions. It is also important to note that the current study was correlational, so that no conclusions can be drawn regarding causality of maternal history in relation to infant brain volumes. The current sample was also characterized by relatively high family income and maternal education, which might explain why socioeconomic indicators were not associated with infant brain volumes. It will be important to understand the role of maternal maltreatment experiences on infant brain structure in more varied sociocultural contexts in future work. In addition, measures of maternal childhood maltreatment rely on self-report and may be subject to biased recall, although similar measures have shown good validity in relation to developmental and health outcomes (Felitti et al., 1998; Racine et al., 2018), as well as child protective service reports (Kobulsky et al., 2018). Finally, segmentation of infant brains is challenging, with lower signal to noise, contrast to noise, and spatial resolution relative to anatomy of mature brains. Therefore, subtle differences in structural volumes could be missed. Finally, two important theoretical issues were not addressed in this study and remain frontiers of current neuroscience. First, this study did not assess mechanisms through which maternal childhood maltreatment might affect infant neurobiological development. Second, it remains unclear whether the neurobiological differences observed here act as mechanisms mediating the later social and emotional outcomes associated with maternal childhood maltreatment. Work identifying mechanisms at both levels is a critical need in future work.

### Conclusions

This study adds important specificity to previous work linking mother’s overall childhood maltreatment history to reductions in infant GMV and amygdala volume (Khoury et al., 2021a; Moog et al., 2018). The current results suggest that these intergenerational effects of adversity are differentiated, such that abuse or neglect in the previous generation is linked to differences in the development of specific brain regions in the next generation. Therefore, it will be important that future work distinguish between abuse and neglect in studies examining effects of maternal adversity on the developing brain. However, given the modest sample size, the initial evidence provided in the current study should be followed by future work in larger samples that can probe more complex models that both include other variables of interest, such as infant temperament and additional maternal physical and mental health variables, and that formally test mediational pathways and mechanisms that may account for these reported associations.

The current results in infants under two years of age also underscore both the importance and the feasibility of early identification of mothers with histories of childhood maltreatment. The ACE questionnaire used here is brief and well-suited for screening pregnant women in primary care settings. Notably, a number of evidence-based supportive interventions have demonstrated efficacy in improving outcomes for socially at-risk infants (e.g., Tereno et al., 2017; Yarger et al., 2020). Thus, the effects on infant brain development observed here may be preventable if underlying mechanisms can be identified and targeted by supportive interventions for maltreated women during the perinatal period.

## Data Availability

The data that support the findings of this study are available from the corresponding author upon reasonable request.

## Footnotes

1 Given that R will not run models using the SEM function when the variances of variables are too large, all brain volumes were divided by 1000 and age in days was divided by 100 to reduce the absolute size of the variances. All significant results remained the same when analyzed with SPSS using the variables as originally scaled and either omitting outliers or winsoring them. Thus, all significant results were robust to whether or not FIML was used to estimate missing data and to whether or not the variables were rescaled by dividing by multiples of 10.

2 GMV was not controlled in the analyses of limbic volumes. Increasing evidence indicates that GMV is affected by an array of environmental risk factors, including maternal childhood maltreatment (Khoury Ahtem, et al., 2021; Moog et al., 2018), the predictor of interest in the present report. Thus, we covaried sex and age to control for extrinsic variance related to head size.
